# Monocyte-Derived Dendritic Cells Differentiated in the Presence of Lenalidomide Display a Semi-Mature Phenotype, Enhanced Phagocytic Capacity, and Th1 Polarization Capability

**DOI:** 10.3389/fimmu.2018.01328

**Published:** 2018-06-13

**Authors:** Juan López-Relaño, Beatriz Martín-Adrados, Irene Real-Arévalo, Javier Lozano-Bartolomé, Beatriz Abós, Silvia Sánchez-Ramón, Bárbara Alonso, Manuel Gómez del Moral, Eduardo Martínez-Naves

**Affiliations:** ^1^Departamento de Inmunología, Facultad de Medicina, Universidad Complutense, Madrid, Spain; ^2^12 de Octubre Health Research Institute (imas12), Madrid, Spain; ^3^Departamento de Inmunología, Hospital Clínico San Carlos, Madrid, Spain; ^4^Centro de Investigaciones Biológicas, Madrid, Spain; ^5^Departamento de Biología Celular, Facultad de Medicina, Universidad Complutense, Madrid, Spain

**Keywords:** lenalidomide, dendritic cells, antigen presentation, phagocytosis, NKT cell, T-cells, cytokines

## Abstract

Lenalidomide is an analog of thalidomide, with potent anticancer activity demonstrated in several hematological malignancies. It has immunomodulatory properties, being able to enhance the activation of different types of immune cells, which results in antitumor activities. Dendritic cells (DCs) are pivotal in the immune response, and different immunotherapeutic approaches targeting these cells are being developed. Since little is known about the effect of lenalidomide on DCs, the goal of the present work was to investigate the phenotype and function of human monocyte-derived DCs differentiated in the presence of lenalidomide (L-DCs). Our results showed that L-DCs display a unique phenotype, with increased cell surface expression of some maturation markers such as CD1d, CD83, CD86, and HLA-DR. This phenotype correlates with a lower expression of the E3 ubiquitin-ligase MARCH-I in L-DCs, upregulating the cell surface expression of CD86 and HLA-DR. In addition, immature L-DCs express higher amounts of DC-SIGN on the cell surface than control immature DCs. After LPS stimulation, production of IL-6 and TNF-α was severely decreased, whereas IL-12 and IL-10 secretion was dramatically upregulated in L-DCs, compared to that in the controls. Functionally, L-DCs are more effectively recognized by NKT cells in cytotoxicity experiments. Furthermore, L-DCs display higher opsonin-independent antigen uptake capability than control DCs. Mixed lymphocyte reaction experiments showed that L-DCs could stimulate naïve CD4 T-cells, polarizing them toward a predominant Th1 phenotype. In summary, DCs derived from monocytes in the presence of lenalidomide present a semi-mature phenotype, increased phagocytic capacity, reduced production of proinflammatory cytokines, and the ability to polarize T-cells toward predominant Th1-type responses; these are qualities that might be useful in the development of new immunotherapeutic treatments.

## Introduction

Lenalidomide (Revlimid^®^) is a derivative of thalidomide (Thalomid^®^) (1) approved for the treatment of multiple myeloma (MM), myelodysplastic syndromes associated with 5q cytogenetic abnormalities, and mantle cell lymphoma (2). Lenalidomide exerts direct tumoricidal activity as well as stimulating antitumor immune responses (3). It can also mediate some of its antitumoral and immunomodulatory activities through the action of the cullin-RINGE3 ubiquitin ligase complex CRL4^CRBN^ (4), promoting the degradation of certain substrates, such as the transcription factors Ikaros (IKZF1) and Aiolos (IKZF3) (4), as well as the casein kinase 1A1 (CK1a) (5). Lenalidomide is able to inhibit proliferation (6) and induce the apoptosis of cancer cells (7, 8). It also interferes with the stromal support of tumor cells by blocking the production of cytokines and growth factors necessary for the cancer cells’ survival (9). It also inhibits the expression of adhesion molecules, impairing the physical interaction between stromal and cancer cells (10, 11) and inhibits the expression of angiogenic factors (12). Furthermore, lenalidomide exerts a wide variety of immunomodulatory actions, which can contribute to tumor control. The drug inhibits the production of cytokines such as TNF-α, IL-6, or IL-1β, whereas it promotes the release of IL-2, IL-10, IL-8, and INF-γ (13, 14). It also enhances the antitumor activity of NK cells and T-cells, the response to which is an increase in their proliferation, activation, cytotoxic capability (15), and production of cytokines such as IL-2 and IFNγ (16–18). In addition, it has been described that lenalidomide inhibits the proliferation and function of T-regulatory cells (19); which might interfere with the antitumor activity of effector T-cells. Furthermore, NKT cells, which are specialized T lymphocytes that recognize glycolipidic antigens presented by CD1d molecules, are activated by lenalidomide, which enhances the response of these cells against tumors (20).

Dendritic cells are crucial for the activation of immune responses mediated by T cells (21), which explains why these cells are the target of many experimental approaches focused on the development of antitumor vaccines (22, 23).

Lenalidomide has been found to be useful to enhance immune responses in dendritic cell-based immunotherapy animal models of colon cancer (24, 25) and MM (26). Moreover, it has also been recently reported that lenalidomide can enhance the recovery and functions of monocyte-derived DCs generated from MM patients (24, 25). Since the effect of lenalidomide on DCs, especially from healthy people, has not been investigated in depth, the goal of the present work was to investigate how the drug affects the three main functions of DCs: capture and presentation of antigens, activation of T lymphocytes, and production of cytokines. We found that human monocyte-derived DCs from healthy blood donors, differentiated in the presence of lenalidomide (L-DCs), display unique phenotypic and functional features that can be useful to manipulate and enhance immune responses.

## Materials and Methods

### Culture Medium and Reagents

The following culture media were used: RPMI 1640 medium (Gibco), complete medium, cRPMI (RPMI 1640), 1% antibiotic/antimycotic solution (Invitrogen Life Technologies), and 10% heat-inactivated (60 min, 57°C) fetal calf serum (FCS Harlan Sera-Lab). ACK lysis buffer (0.5 M NH_4_Cl, 10 mM KHCO_3_, and 0.1 nM Na_2_EDTA at pH 7.4) was used to eliminate red blood cells (RBCs) from buffy coats in the peripheral blood samples. A glycolipid antigen α-GalCer analog (PBS-57, a gift from Paul B. Savage) was used for NKT cell expansion, as well as in the cytotoxicity assays. Cytokines: recombinant human interleukin 4 (rhIL-4), recombinant human interleukin 2 (rhIL-2), and recombinant human granulocyte-macrophage colony-stimulating factor (rhGM-CSF) (Immunotools).

### Immunomodulatory Drug

Lenalidomide (CC-5013, Revlimid) was obtained from Celgene Inc. and freshly dissolved in dimethyl sulfoxide (DMSO) to achieve a final concentration of 2 mM. The compound was further diluted directly into the culture media at the required concentrations. The final concentration of DMSO in all experiments was ≤0.01%, and all treatment conditions were compared with those of the vehicle control (DMSO 0.01%).

### Blood Samples

Buffy coat peripheral blood samples from healthy blood donors were obtained from the Blood Transfusion Service of Madrid. Approval was obtained by the Ethical Committee of Clinical Research of Hospital Clínico de San Carlos (Madrid), reference number: 17/505-E.

### Monocyte Isolation and Dendritic Cell (DC) Differentiation

Peripheral blood mononuclear cells (PBMCs) were isolated from healthy blood donors by Ficoll-Hypaque (1.077 g/ml) density gradient centrifugation. CD14^+^ monocytes were isolated from PBMCs using immunomagnetic bead selection with CD14 microbeads (Miltenyi Biotec). In order to generate monocyte-derived DCs, CD14^+^ cells were cultured at a density of 1.5 × 10^6^ cells/ml in cRPMI containing 1,000 U/ml rhIL-4 and 1,000 U/ml rhGM-CSF, in the presence or absence of 1 µM lenalidomide for 5 days, with cytokine and lenalidomide addition every second day. To induce maturation, cultures of monocyte-derived DCs were supplemented with 5 µg/ml LPS for 48 h. No lenalidomide was added during the maturation phase or in the co-culture experiments with T-cells.

### Human iNKT Cell Expansion, Isolation, and Cytotoxicity Assays

iNKT cells were expanded from total PBMCs by treatment with 100 U/ml rhIL-2 in the presence of PBS-57 (100 ng/ml) for 2 weeks. After that, iNKT cells (Vα24^+^ Vβ11^+^ cells) were positively selected by magnetic separation (Miltenyi Biotec) and analyzed using the specific anti-iNKT antibody. Purity of the iNKT cells was always higher than 95% (Figure S1 in Supplementary Material). The purified iNKT cells were used as effector cells in cytotoxicity experiments using DCs, previously loaded with PBS-57, as targets. Cytotoxicity was measured with the non-radioactive Cytotoxicity Detection Kit LDH (Roche), according to the manufacturer’s instructions. The percentage of specific lysis was calculated as follows: 100 × (experimental value − spontaneous release value)/(maximum release value − spontaneous release value). Experimental value = the value obtained with α-Galcer. Spontaneous release = Co-culture (DC:NKT) without α-GalCer. Maximum release = DC treated with detergent, in this case, Triton X-100.

### Antibodies and Flow Cytometry Analysis

Primary unconjugated antibodies used in this study were anti-human CD1a clone OKT6 culture supernatant (from American Type Culture Collection), anti-human HLA-I clone W6/32, anti-human DC-SIGN (27) (a gift from Dr. Angel Corbí). ICAM-I IgG supernatant (HV5/3) and VCAM-I supernatant, a gift from Dr. Sánchez-Madrid. Fluorescein-coupled polyclonal goat anti-mouse IgG (H + L; Caltag Laboratories) was used as the secondary antibody in FACS analysis. The following conjugated antibodies were used: anti-human CD1d-PE, anti-human HLA-DR-PE, anti-human CD80-PE, anti-human CD86-FITC, anti-human CD25-APC, anti-human CD83-APC (all from BD Biosciences), anti-human iNKT-PE (Miltenyi Biotec), and anti-human CD8-PECy7 (Beckman Coulter), anti IFNγ-AF488 (BD Biosciences), anti FoxP3-AF488 (Biolegend). Flow cytometry was performed following standard protocols on a BD FACSCalibur cytometer. Graph bars summarizing flow cytometry data are expressed as relative mean fluorescence intensity (MFI), which is defined as the MFI of any sample divided by the MFI of the immature dendritic cells (iDCs).

For intracellular staining, cells were fixed with PBS-PFA 4% for 20 min, then, the cells were washed twice in PBS containing 0.1% saponin (Sigma-Aldrich), incubated with the desired antibodies in 100 ml of PBS containing 1% saponin for 30 min at room temperature, and washed with PBS/0.1% saponin buffer.

### Real-Time Quantitative RT-PCR

Quantitative PCR was performed using real-time PCR (ABI PRISM 7700; Applied Biosystems): 40 cycles at 95°C for 15 s and 60°C for 60 s using the Roche UPL System assay. The comparative RQ = 2^−ΔΔct^ method was used to quantify the transcripts and normalize the expression levels of the tested genes to those of GAPDH. Probes were designed using the Universal Library Probe system Roche.

### Phagocytic Assays

Dendritic cells differentiated in the presence (L-DCs) or absence (DCs) of lenalidomide were incubated with fluorescent-labeled zymosan or with IgG-opsonized sheep RBCs for 20 min on ice and subsequently in a 37°C prewarmed bath for 30 min. After being placed back on ice for 10 min, the cells were pretreated for 1 min with ice-cold quenching solution for suppressing the fluorescence of the yeast cells attached to the cell membrane. RBCs attached to the cell membrane were lysed by adding distilled water for 5 s, followed by PBS addition to recover the isotonic conditions. Phagocytic index was measured in a FACScalibur by the MFI.

### Proliferation Assays

L-matured DCs (mDCs) or mDCs were used as stimulators for allogeneic naïve or autologous CD4^+^ T-cells isolated from human peripheral blood using MACS (Miltenyi Biotec) and subsequently labeled with 2 µM CFSE (Sigma-Aldrich). The co-cultures were performed in 96-well flat-bottom culture plates. Lymphocyte proliferation was assessed by the CFSE dilution assay using CD3^+^ cells on day 5. Blocking experiments were performed in the presence of anti-human IL-10 monoclonal antibody (Invitrogen).

Peptide pools for HLA class I (CEF Pool) or class II (CEFT MHC-II pool) representing multiple antigens (JPT Peptide Technology) were used to assess specific T-CD8 or T-CD4 cell responses. The CEF pools consist of mixed peptides each corresponding to defined HLA class I- or class II-restricted T-cell epitopes that were selected on the basis of a high frequency of responsiveness of PBLs in healthy volunteers.

IL-4, IL-10, and IFNγ were measured in the supernatants of the cultures using the BD bioscience CBA system for flow cytometry.

### Cytokine Detection by Enzyme-Linked Immunosorbent Assay (ELISA)

Supernatants of the cell cultures were harvested and stored at −80°C until they were thawed, and all were run in the same batch in ELISA experiments to quantify cytokine secretion. Production of IL-12p70, TNF-α, IL-10, and IL-6 was analyzed using pairs of commercially available monoclonal antibodies for ELISA (Invitrogen), following the manufacturer’s protocol.

### Western Blotting

Cells were lysed in RIPA buffer (150 mM Sodium Chloride, 1% NP-40, 5% sodium deoxycholate, 0.1% SDS, 20 mM Tris pH 7.5) supplemented with Protease Inhibitor Cocktail (Sigma-Aldrich) and phosphatase inhibitors PhosSTOP™ (Roche). The proteins were resolved by electrophoresis on a 12% polyacrylamide gel and transferred to PVDF membranes (Merck Millipore) at 4°C. Membranes were blocked using 1% skimmed milk powder and 5% BSA in TBS and incubated overnight at 4°C with the following primary antibodies: anti-pERK 1:1,000 (Cell Signaling), p651:500 (Cell Signaling), total ERK 1:1,000 (Cell Signaling), total p651:500 (Cell Signaling), and tubulin 1:1,000 (Sigma-Aldrich). The following secondary antibodies were added and the membranes were then incubated for 1 h at RT: donkey anti-rabbit IRDye680RD 1:10,000 and donkey anti-mouse IRDye800CW 1:10,000 (LI-COR Biosciences). The membranes were scanned using the Odyssey Infrared Imaging System (LI-COR Biosciences).

### Statistical Analysis

Mean values were compared using the unpaired Student’s *t-*test. All statistical analyses were performed with Statgraphics or Excel software. Statistically significant differences were represented as: *p < 0.05, **p < 0.03, and ***p < 0.01.

## Results

### Monocyte-Derived DCs Differentiated in the Presence of Lenalidomide Display a Unique Semi-Mature Phenotype

**Figure 1:**
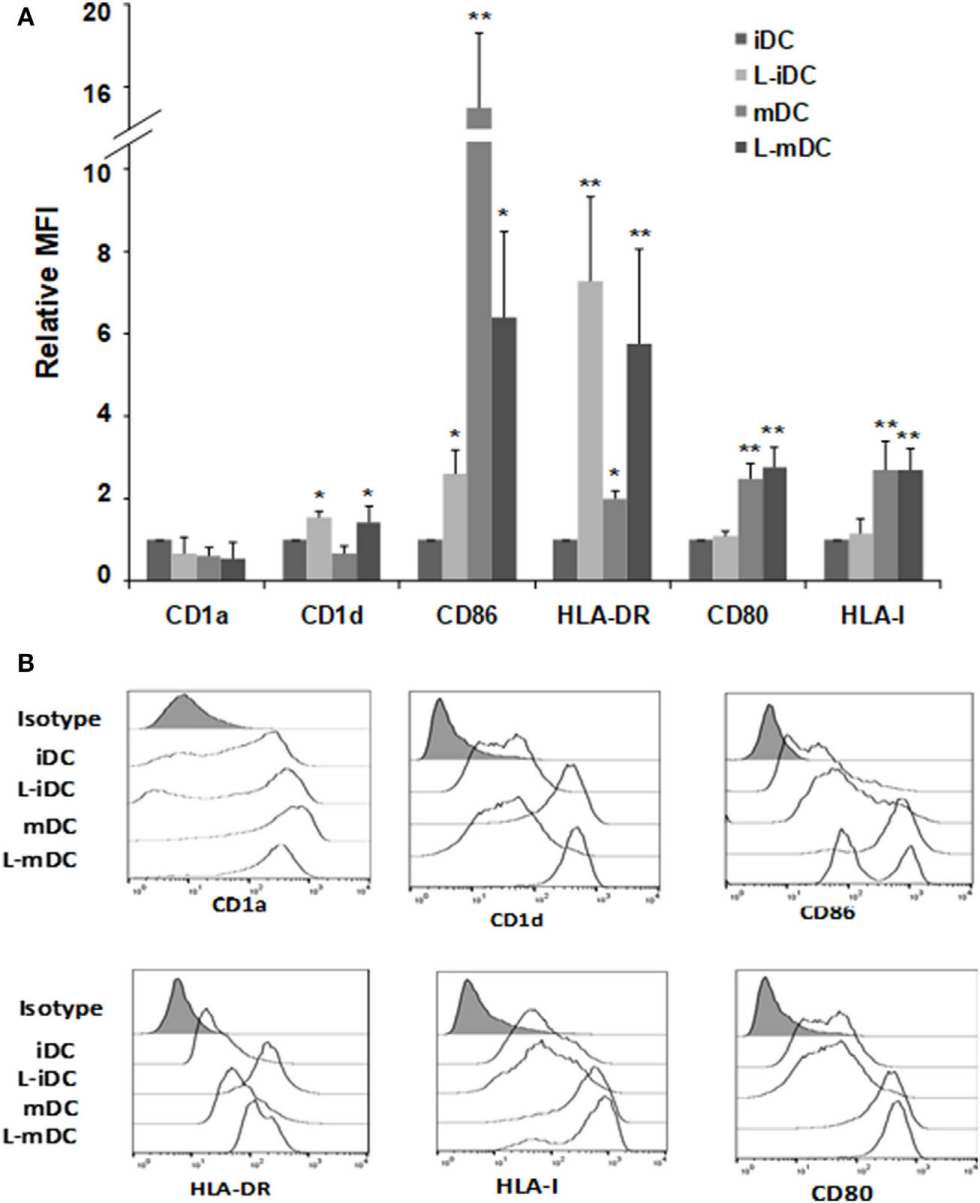
Monocyte-derived dendritic cells (DCs) differentiated in the presence of lenalidomide (L-DCs) increase their surface expression of CD1d, CD86, and HLA-DR molecules. Expression of different cell surface markers in monocyte-derived DCs was measured by flow cytometry. **(A)** Data shown are the relative mean fluorescence intensity (MFI) ± SDs from 12 independent experiments, comparing L-iDCs, matured DCs (mDCs), or L-mDCs to iDCs. **(B)** Histograms from a representative experiment are shown (*p < 0.05, **p < 0.03).

**Figure 2:**
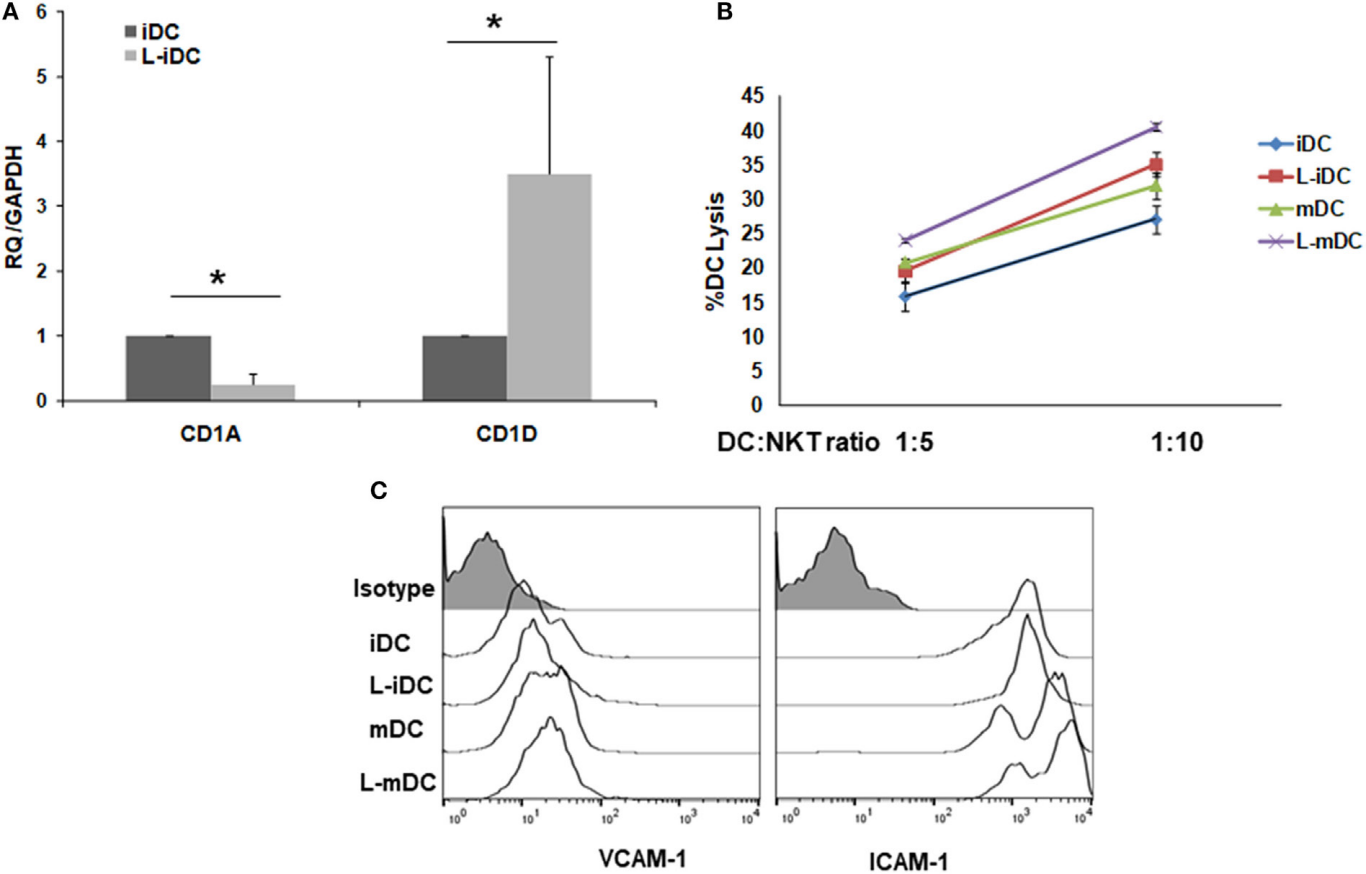
CD1A and CD1D genes are counter-regulated in L-iDCs. The increase in CD1D expression results in enhanced recognition of L-iDCs by NKT cells. **(A)** Relative quantification of *CD1A* and *CD1D* gene transcript levels in control dendritic cells (DCs) and L-DCs. Data (mean ± SDs) from four independent experiments are shown. **(B)** α-GalCer-loaded L-iDCs or iDCs were co-cultured with previously purified NKT cells at two different ratios for 4 h. A lactate dehydrogenase detection kit was used to measure the lysis of DCs. The average of four independent experiments is shown (*p < 0.05). **(C)** Expression of VICAM-I and ICAM-I on DCs. A representative from four experiments is shown.

**Figure 3:**
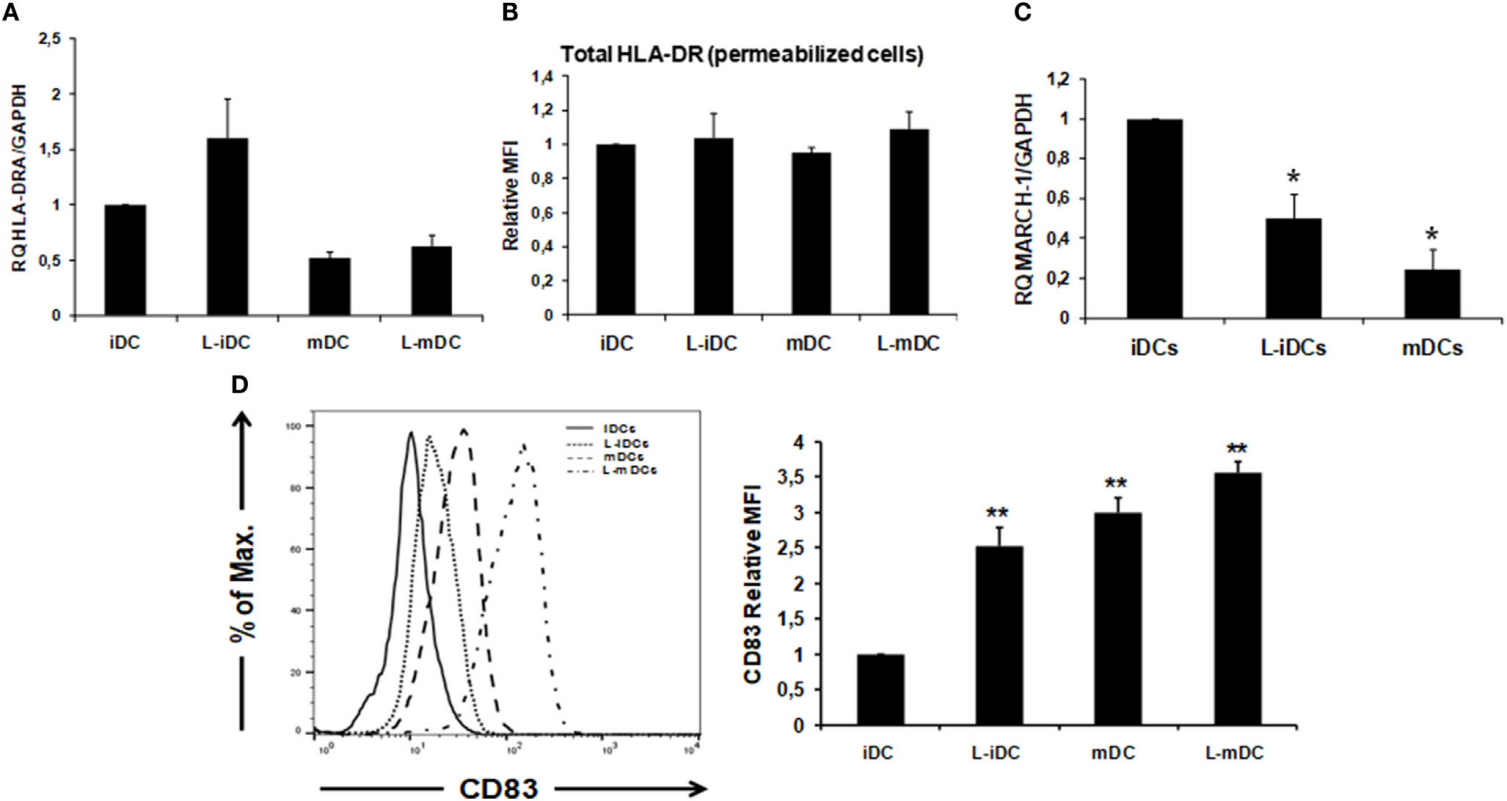
HLA-DR cell surface expression correlates with an increase in CD83 expression and a downregulation of MARCH-1. **(A)** Relative quantification of *HLA-DRA* gene transcription in control and L-DCs. Data (mean ± SDs) from three independent experiments are shown. **(B)** Total HLA-DR protein (extra +intracellular) levels were assessed by flow cytometry in lenalidomide-treated and untreated DCs. Data from three independent experiments are shown as the relative mean of fluorescence intensity (MFI) ± SDs from permeabilized cells (*p < 0.05; **p < 0.03). **(C)** MARCH-1 mRNA is downregulated in L-DCs. Q-PCR data from four independent experiments are shown as mean gene expression levels relative to the expression of GAPDH (*p < 0.05; **p < 0.03). **(D)** CD83 surface expression is upregulated in DCs differentiated in the presence of lenalidomide, as measured by flow cytometry. Left: the histogram shows a representative experiment. Right: relative MFI ± SDs from six independent experiments.

Peripheral blood monocytes obtained from healthy blood donors were differentiated into iDCs by treatment with GM-CSF plus IL-4 in the presence or absence of lenalidomide. Different concentrations of lenalidomide were used in order to test its toxicity. In the presence of 0.5 and 1 µM lenalidomide, the viability of the cells was higher than 95% after 5 days in culture, whereas at the concentration of 2 µM, the viability was 90%; at lenalidomide concentrations higher than 8 µM, the cell viability was less than 50% (results not shown). Thus, in all further experiments, we decided to use lenalidomide at a concentration of 1 µM. Flow cytometry analysis revealed that iDCs differentiated from monocytes in the presence of lenalidomide (L-iDCs) displayed a distinct phenotype when compared to control cells (iDCs). L-iDCs showed a semi-mature array of markers with increased surface expression of the antigen-presenting molecules CD1d and HLA-DR, as well as the co-stimulatory molecule CD86. In contrast, their expression levels of the HLA class I, CD1a, and CD80 molecules were similar to those observed in case of control iDCs (Figure 1). Next, we studied the phenotype of the cells after maturation with LPS for 48 h. In the control, mDCs, we observed, as expected, upregulation of HLA class I, HLA-DR, CD86, and CD80. In the case of mature cells exposed to lenalidomide (L-mDCs), CD80 and CD86 increased their surface expression upon LPS exposure, whereas no significant upregulation of HLA-DR and CD1d was observed, suggesting that these markers were already at their highest level in L-iDCs. In order to investigate the mechanism underlying these phenotypic observations, we decided to analyze gene expression by RT-PCR. We found that mRNA for *CD1D* was upregulated in L-iDCs, whereas that for *CD1A* was downregulated in these cells, when compared to control iDCs (Figure 2A). Next, we carried out cytotoxicity experiments using DCs as targets of autologous NKT cells. Our results showed that NKT cells recognized L-iDCs more efficiently than the control iDCs, as well as L-mDCs than mDCs, indicating that the enhanced expression of CD1d on L-iDCs is functionally relevant (Figure 2B). We observed no changes in the expression of adhesion molecules that are involved in the immunological synapsis during the T cell-mediated cytotoxicity, VCAM-I and ICAM-I, on L-DCs, compared to DCs (Figure 2C). In RT-PCR experiments, we found a slight upregulation of *HLA-DR* gene expression in L-iDCs, when compared to control iDCs, together with a subtle but statistically significant downregulation of the HLA-DR gene in both L-mDCs and mDCs, when compared to iDCs (Figure 3A). Interestingly, the total amount of HLA-DR protein on permeabilized cells, which was measured by flow cytometry, was similar in the control as well as in both the lenalidomide- and LPS-treated DCs (Figure 3B), suggesting that posttranslational mechanisms are responsible for the enhanced expression of HLA-DR observed on the L-DC surfaces. We also performed experiments to evaluate the effect of lenalidomide during the maturation phase by adding the drug to control iDCs at the same time as LPS. In this case, no phenotypic differences were observed on comparing the control cells with the lenalidomide-exposed cells (results not shown).

### HLA-DR and CD86 Protein Expression on the Cell Surface Correlates With MARCH-1 and CD83 Levels

Expression of CD86 and HLA-DR on the surface of DCs is post translationally regulated by the action of the E3 ubiquitin-ligase MARCH-I (28, 29). In addition, expression of CD83 has been described to enhance MHC class II and CD86 expression on DCs by opposing MARCH1-mediated ubiquitination and degradation (30). Thus, we decided to analyze the levels of MARCH-I and CD83 in DCs. Our results showed that MARCH-1 mRNA expression was lower in L-iDCs than in control iDCs (Figure 3C). On the other hand, CD83 was upregulated on the surface of L-iDCs, compared to the case for the control DCs (Figure 3D); these variations correlate with HLA-DR and CD86 levels on the L-DC surfaces.

### L-DCs Display Enhanced Opsonin-Independent Antigen Uptake Capability

**Figure 4:**
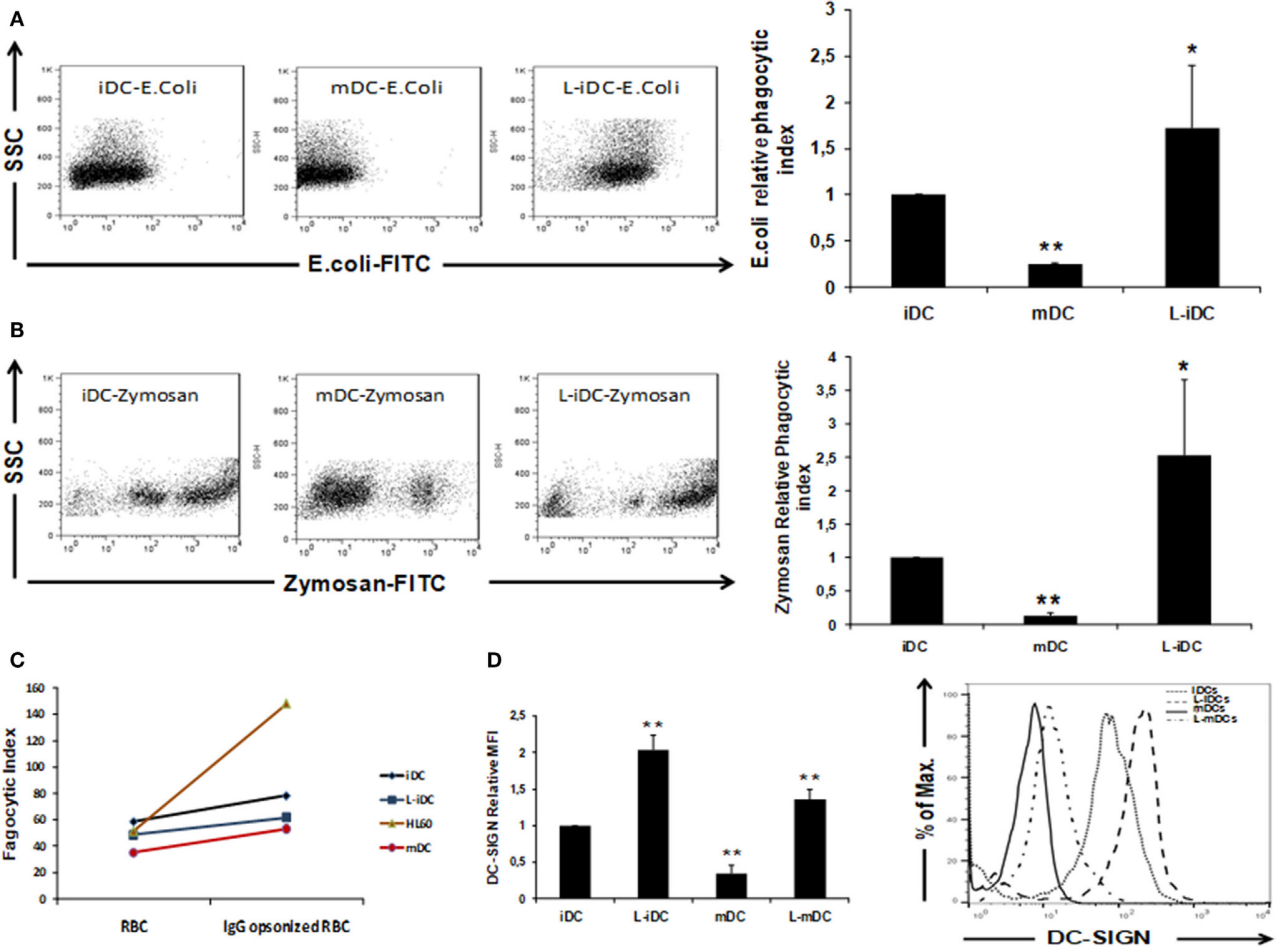
Phagocytosis of *Escherichia coli* and Zymosan is enhanced in L-iDCs. **(A)** Non-opsonized FITC-labeled *E. coli* was phagocytized more efficiently by L-iDCs than control DCs. The number of dendritic cells (DCs) showing green fluorescence was determined by flow cytometry. Left panel shows a Dot Plot with SSC vs FITC fluorescence from a representative experiment. Right panel shows the phagocytic index average of five independent experiments. Phagocytic index was calculated as (% positive cells × mean channel fluorescence). **(B)** Non-opsonized FITC-labeled Zymosan was phagocytized more efficiently by L-iDCs than control DCs. The number of DCs showing green fluorescence was determined by flow cytometry. Left panel shows a Dot Plot with SSC vs FITC fluorescence from a single experiment. Right panel shows the phagocytic index average of five independent experiments. **(C)** Green-labeled sheep red blood cells previously opsonized with human AB + serum were used to perform phagocytosis assays. HL60 cells were used as a control for opsonization-dependent phagocytosis. The number of DCs showing green fluorescence was determined by flow cytometry. The average of two independent experiments is shown. **(D)** DC-SIGN expression is upregulated on the surface of DCs differentiated in the presence of lenalidomide. Left panel shows the relative mean fluorescence intensity (MFI) ± SDs of three independent experiments. Right panel shows a representative histogram of a DC-SIGN staining (*p < 0.05; **p < 0.03).

Our data showed that L-DCs display a unique semi-mature phenotype. Thus, we decided to investigate how these cells behave from a functional point of view. To this purpose, we analyzed the three main functions of a dendritic cell: antigen uptake, cytokine production, and T-cell stimulation capability. Normal iDCs are characterized by high antigen uptake capacity, whereas in mDCs, the phagocytic function is severely reduced (31). In phagocytosis experiments, we found that non-opsonized *Escherichia coli* and zymosan uptakes (Figures 4A,B) were higher in L-iDCs than in control iDCs. In contrast, no differences were observed in opsonization-dependent phagocytosis (Figure 4C). Furthermore, we observed (Figure 4D) that differences in the phagocytosis of non-opsonized particles correlated with the cell surface expression of DC-specific ICAM-grabbing non-integrin DC-SIGN (CD209), a major non-opsonic receptor for zymosan in human DCs.

### L-DCs Exhibit a Differential Cytokine Profile

**Figure 5:**
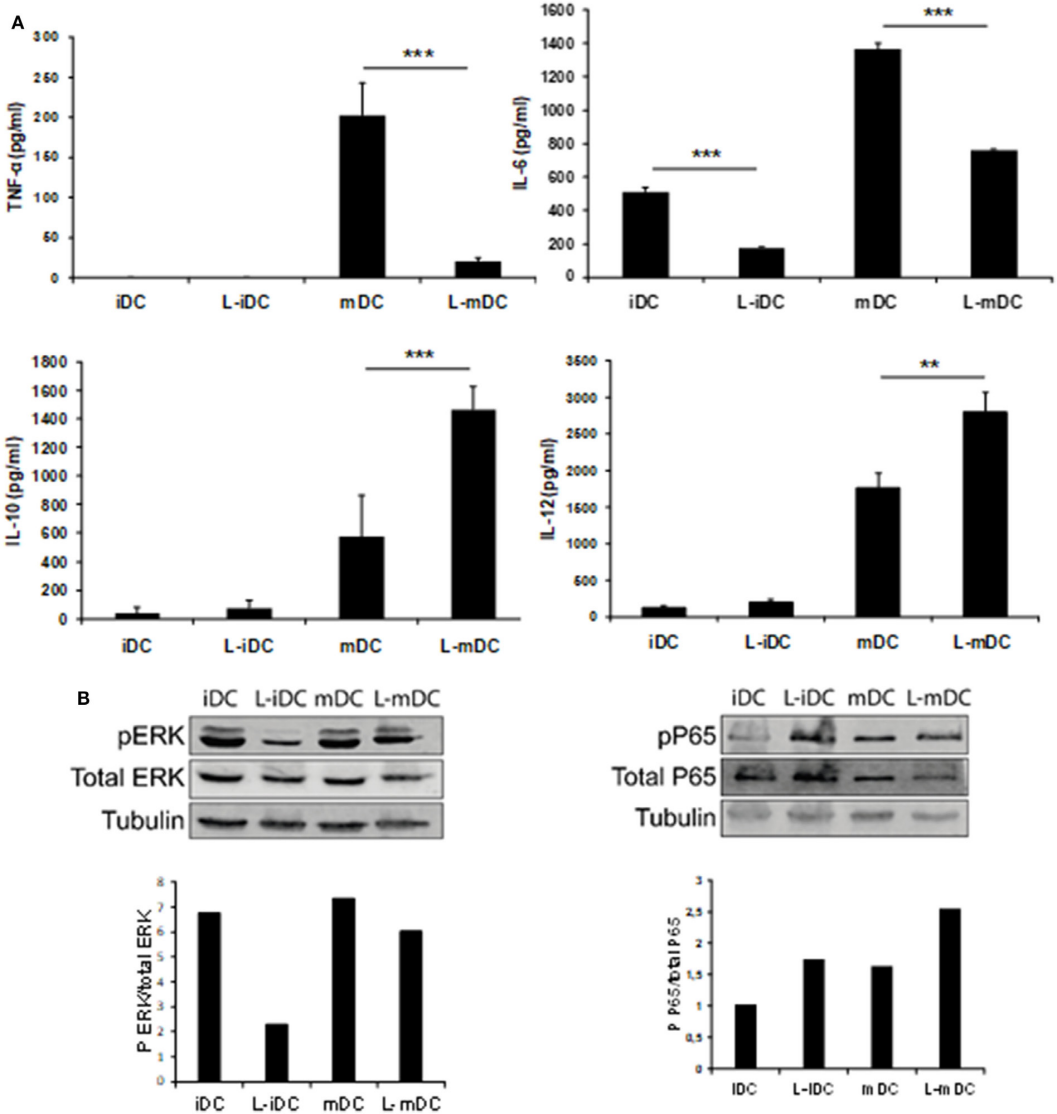
L-mDCs showed impaired production of IL-6 and TNF-α as well as enhanced secretion of IL-10 and IL-12. **(A)** Enzyme-linked immunosorbent assay quantification of secreted cytokines by dendritic cell cultures stimulated with LPS for 48 h. Average cytokine concentration from four independent experiments ± SDs is shown (**p < 0.03; ***p < 0.01). **(B)** Western blotting of whole cell lysates from immature dendritic cells (iDCs), matured DCs (mDCs), L-iDCs, and L-mDCs. The blots were probed with antibodies to total ERK, phospho-ERK, total NFκB, or phospho-NFκB.

**Figure 6:**
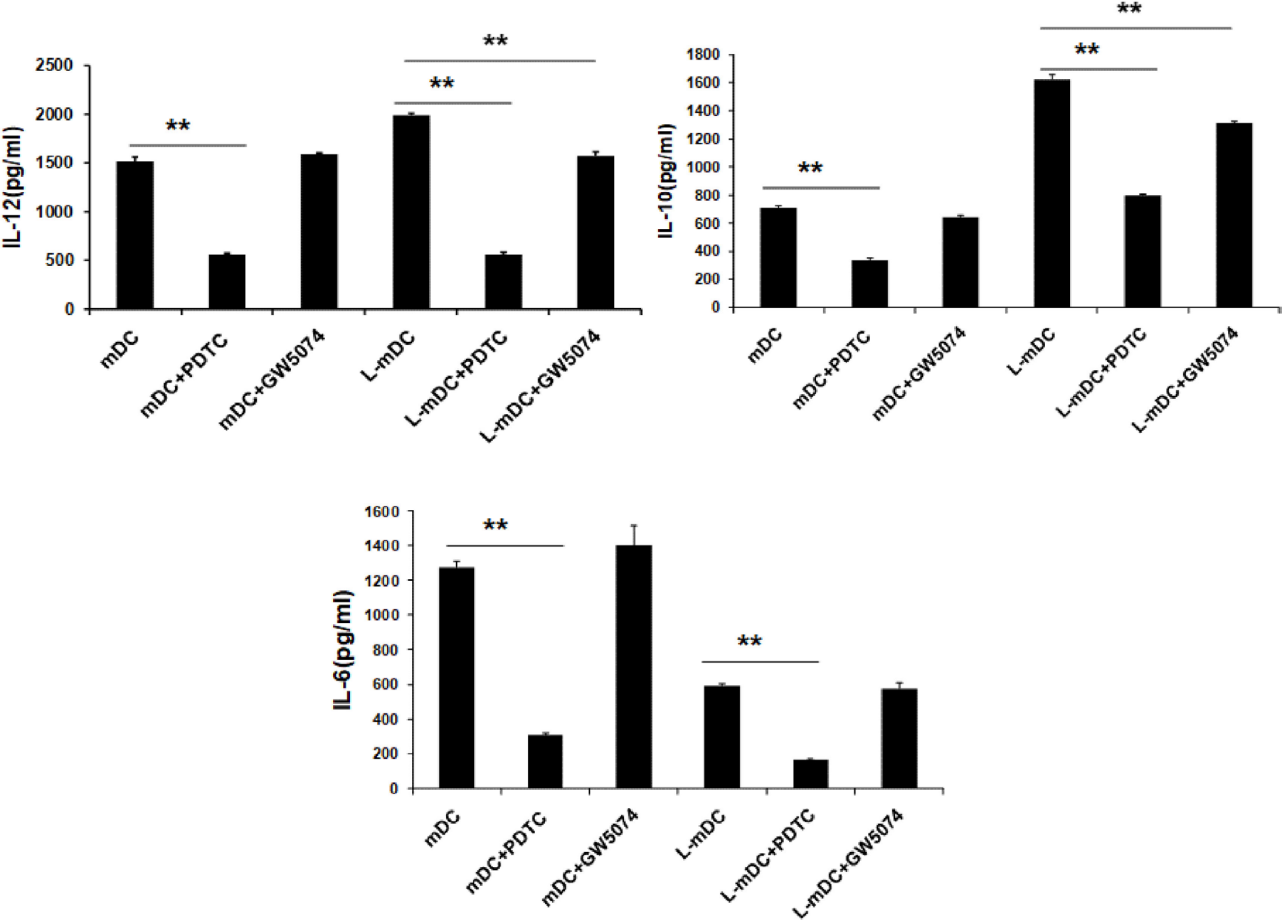
Upregulation of IL-10 and IL-12 in L-mDCs is dependent on Raf-1 activity. DCs were stimulated with LPS and secretion of cytokines was measured by enzyme-linked immunosorbent assay. DCs were treated with GW5074, a Raf-1 inhibitor, or PDTC, an NF-κB inhibitor, as a control. Average cytokine measure of three independent experiments ± SD is shown (**p < 0.03).

Secretion of TNF-α, IL-6, IL-10, and IL-12 was assessed by ELISA analysis of culture supernatants from control iDCs, L-iDCs, mDCs, and L-mDCs. We found (Figure 5A) that the production of TNF-α by L-mDCs was dramatically reduced in comparison with that by control mDCs. In addition, the capacity to secrete IL-6 was severely impaired in L-iDCs, compared to control iDCs, this was also the case for L-mDCs, compared to the mDCs, suggesting that L-DCs have an anti-inflammatory profile. This was correlated with a reduced level of phosphorylated p-ERK and increased phosphorylation of the NFκB-p65 subunit in both L-iDCs and L-mDCs, when compared to the case for iDCs and mDCs, respectively (Figure 5B). Interestingly, the production of both IL-12 and IL-10 was enhanced in the case of L-mDCs, compared to that in control mDCs (Figure 5A). This was unexpected, taking into account the pro-inflammatory nature of IL-12 and the fact that IL-10 and IL-12 are usually counter-regulated (32). Simultaneous upregulation of IL-10 and IL-12 production by DCs may occur when DC-SIGN triggers the activation of the serine threonine kinase Raf-1, which results in enhanced transcription rates of both IL-10 and IL-12 (33). Since we were stimulating L-DCs, which express very high levels of DC-SIGN on their surfaces, through TLR4 with LPS, we hypothesized that a crosslink between both receptors could explain the cytokine profile observed. To test this hypothesis, we measured the cytokine production in response to LPS of cells treated with the specific Raf-1 inhibitor GW5074. As shown in Figure 6, the levels of IL-12 and IL-10 produced by L-mDCs treated with GW5074 were significantly reduced, when compared to untreated cells. In contrast, no effect was observed when we compared control mDCs treated or untreated with GW5074. Moreover, the production of IL-6 was not altered by the treatment of L-mDCs with the Raf-1 inhibitor, underlining the specificity of the phenomenon observed in the case of IL-10 and IL-12. Collectively, these results strongly suggest that the simultaneous upregulation of IL-10 and IL-12 could be related to the activation of Raf-1in L-DCs.

### L-DCs Have the Capacity to Induce the Differentiation of T-Cells With a Predominant Th1-Phenotype

**Figure 7:**
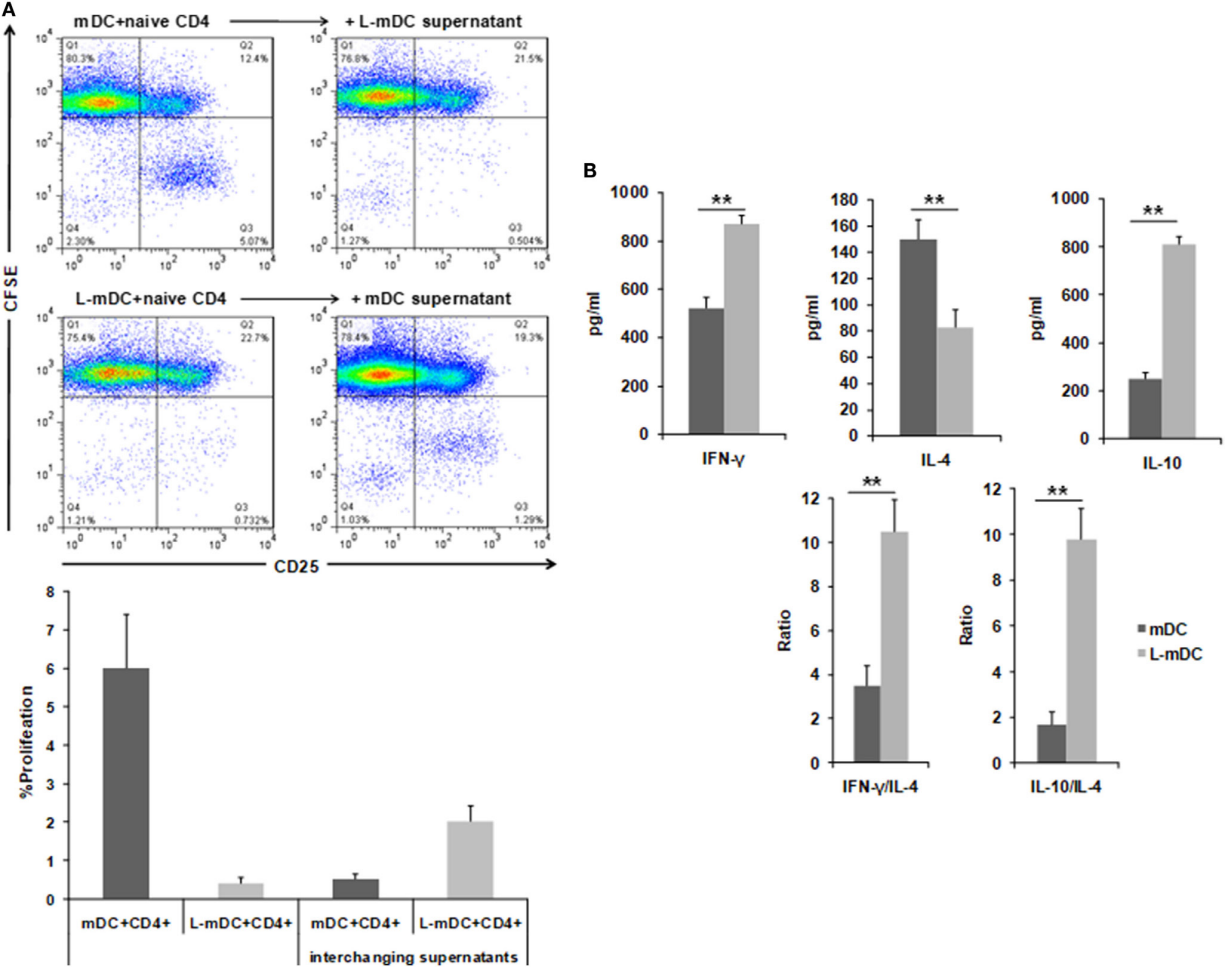
L-DCs induce naïve CD4^+^ T-cell activation and Th1 polarization. Mixed lymphocyte cultures were performed with DCs and allogeneic CD4^+^ naïve T-cells stained with CFSE. **(A)** CD3^+^CD4^+^ T-cells were gated after 5 days of co-culture and then CFSE and CD25 expression was analyzed. Upper left panel shows CD4^+^ T-cell proliferation (CD25^+^, CFSE-cells) after being co-cultured with control DCs. The upper right panel shows the same experiment, but was performed by adding supernatants from L-mDCs. Lower left panel shows that all the CD4^+^CD25^+^ T-cells are CFSE^+^. The lower right panel shows the same experiment, but was performed with supernatants from control mDCs. A representative of four experiments is shown. **(B)** Th1 vs Th2 cytokine profiles were assessed by enzyme-linked immunosorbent assay quantification of IFNγ, IL-4, and IL-10 in the mixed lymphocyte culture supernatants (**p < 0.03).

In order to determine the T-cell stimulatory capacity of the L-DCs, mixed leukocyte reactions (MLR) were carried using allogeneic naïve CD4^+^ T-cells as responders. As shown in Figure 7, when control DCs were used as stimulators, a population of CD4^+^ T-cells become activated, as indicated by expression of CD25, and a subset of these cells proliferate, as indicated by the CFSE dilution assay. In contrast, when L-DCs were used as stimulators, we observed the activation of CD4^+^ allogeneic cells, as indicated by CD25 upregulation. However, no proliferation was observed. Furthermore, when supernatants from previous L-DCs-naïve CD4^+^ T-cells cultures were added to the MLR assay using control DCs, we observed an inhibition of the CD4^+^ T-cell proliferation, which suggests that some soluble factor(s) secreted by the L-DCs inhibits T-cell proliferation. We measured the amount of INF-γ, IL-4, and IL-10 in the supernatants of the co-culture experiments and found that the levels of INF-γ and IL-10 were higher in cultures of L-DCs, whereas IL-4 was more abundant in the experiments in which we used control DCs as stimulators. This suggests that T-cells activated by L-DCs have a predominant Th1 polarization phenotype.

**Figure 8:**
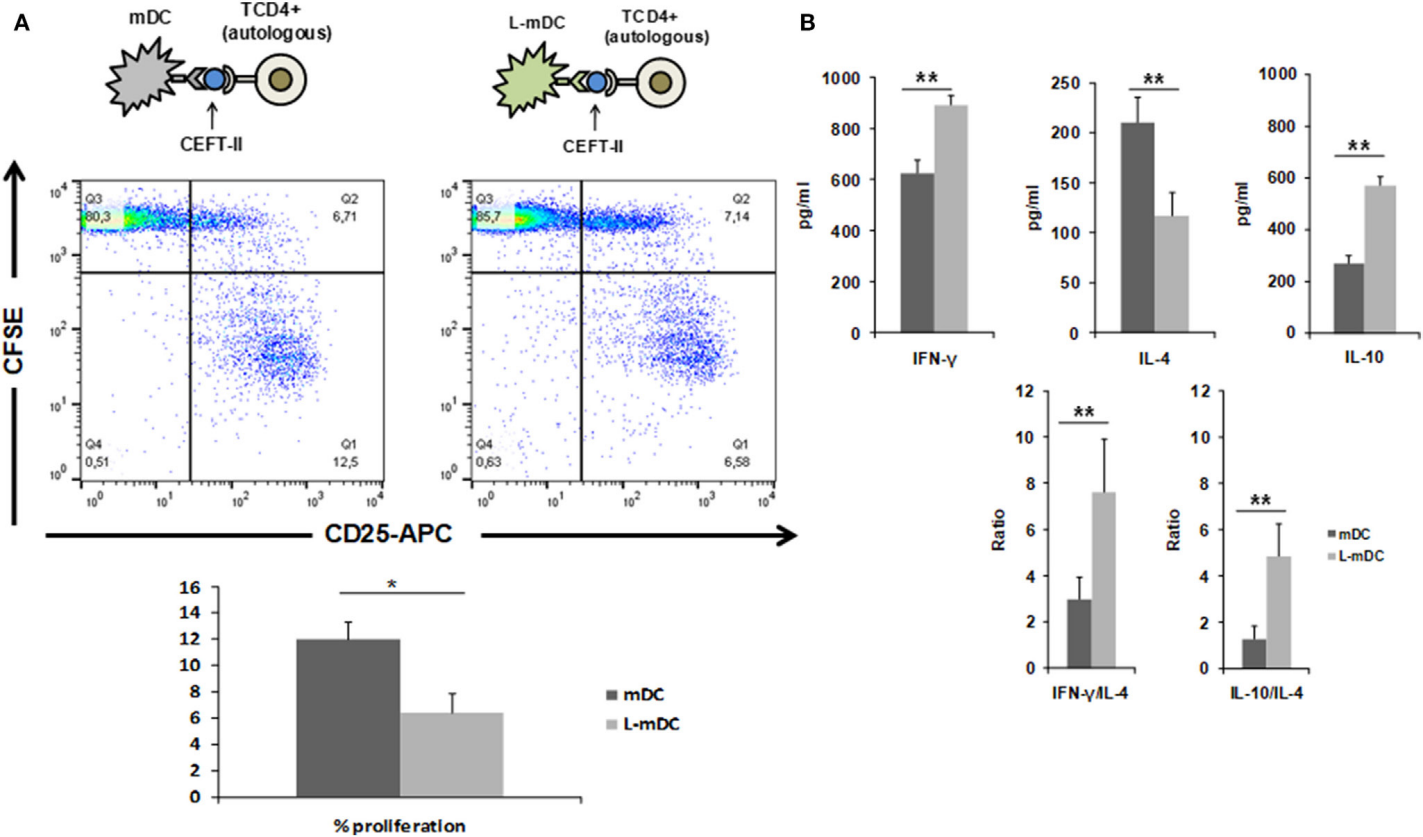
L-matured DCs (mDCs) induce CD4^+^ antigen-specific T-cell responses that are characterized by Th1 bias and diminished proliferation. Mixed lymphocyte cultures were performed with CEFT MHC-II pool preloaded DCs and autologous CD4^+^ T-cells stained with CFSE. **(A)** CD3^+^CD4^+^ cells were gated after 5 days of co-culture and then, CFSE and CD25 expression was analyzed. A representative of four experiments is shown. **(B)** Th1 vs Th2 cytokine profiles were assessed by enzyme-linked immunosorbent assay quantification of IFNγ, IL-4, and IL-10 in the culture supernatants (*p < 0.05; **p < 0.03).

**Figure 9:**
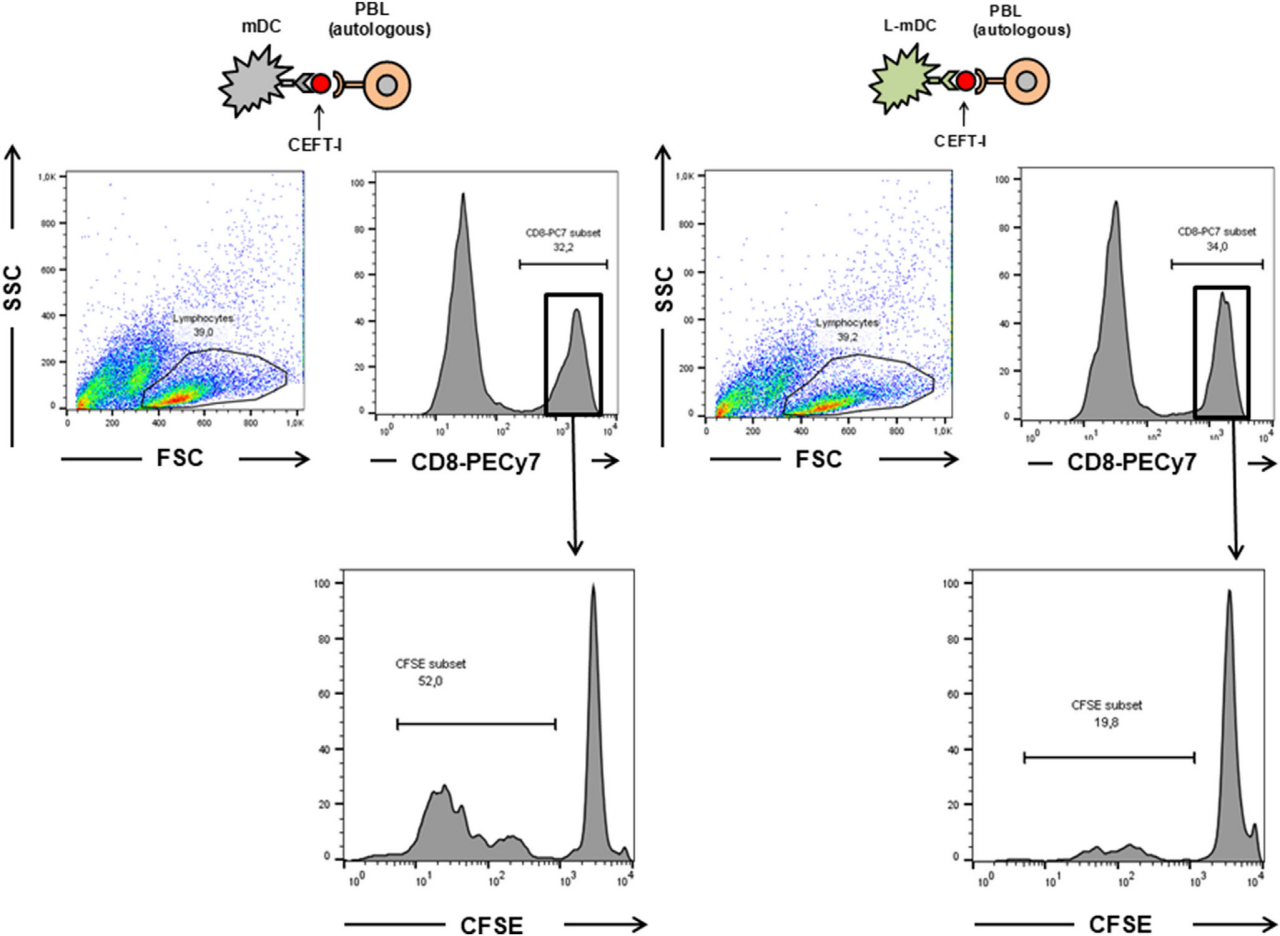
The proliferation of CD8^+^ antigen-specific T-cells is reduced upon L-mDC stimulation. Autologous PBLs were co cultured with mDC or L-mDC previously incubated with CEFT-I peptide pool. The upper panel shows the region gated for the analysis of CFSE dilution. The lower panel shows the proliferation (CFSE dilution) of the PBLs after 14 days of co-culture.

**Figure 10:**
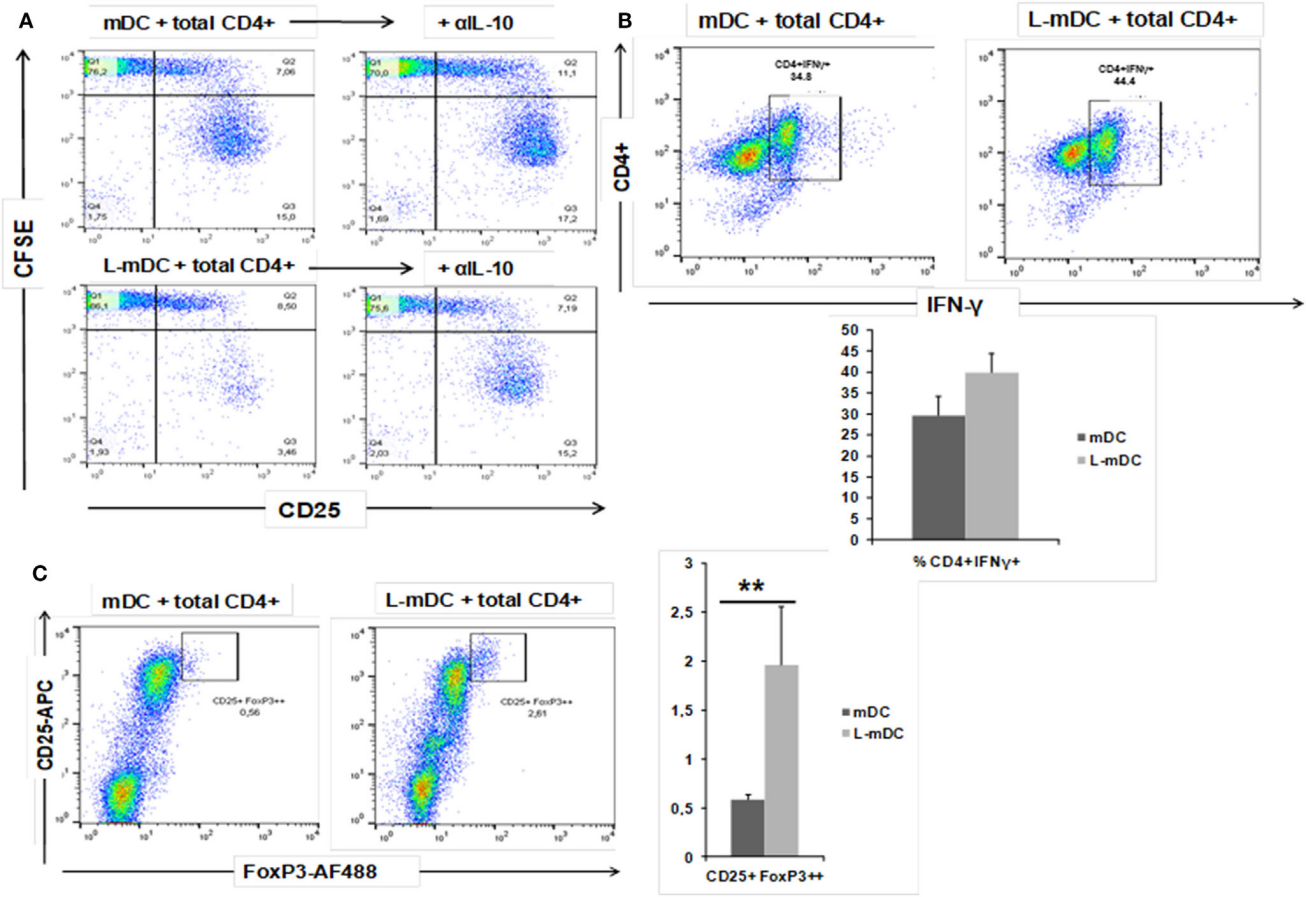
L-mDC co-cultured with heterologous total CD4^+^ T-cells reveal IL-10-dependent proliferation suppression, increased IFN-γ production, and Treg expansion. **(A)** L-mDC proliferation suppression in total CD4^+^ T-cells mixed co-cultures is IL-10-dependent. Antigen-presenting cells, mDCs, or L-mDCs, were co-cultured with heterologous total CD4^+^ T-cells (ratio 1:10) for 5 days in the presence of an isotype control (left) or an anti-IL-10 (5 µg/ml) monoclonal antibody (right). **(B)** Total CD4^+^ T-cells produce higher levels of IFN-γ when stimulated with L-mDC. After 5 days of co cultures, cells were stimulated for 4 h with PMA/Ionomycin in the presence of brefeldin; then, the cells were harvested and intracellularly stained with an anti-IFN-γ monoclonal antibody. **(C)** L-mDCs expand higher numbers of regulatory CD4^+^CD25^high^ FoxP3^high^ T-cells after 5 days of co-culture with total CD4^+^ cells (**p < 0.03).

We also tested the CD4^+^ T-cell antigen-specific stimulation by pre-loading mDCs or L-mDCs with CEFT MHC-II pool. As shown in Figure 8, there is significant reduction in the proliferation of CD4^+^ T-cells co-cultured with L-mDCs and the Th1 profile bias, together with an enhancement of IL-10 secretion, according to the cytokine levels measured in the supernatants. Moreover, we tested the antigen-specific presentation for CD8^+^ T-cells using pre-loaded mDCs or L-mDCs with CEF MHC-I pool in co-cultures with autologous PBLs (Figure 9). Once again, proliferation of T-cells was reduced when L-mDCs were used as stimulators. Our results showed that the proliferation of naïve alloreactive CD4^+^ T lymphocytes was reduced when L-mDCs were used as antigen-presenting cells; this was also the case for autologous peptide-specific memory CD4^+^ as well as CD8^+^ T lymphocytes. These observations correlated with an excess of IL-10 in mDCs culture supernatant, suggesting that this cytokine could be inhibiting the proliferation of T-cells. Therefore, we decided to perform allorecognition experiments using total T-CD4^+^ lymphocytes (that is, including naïve and memory T-cells) as responders (Figure 10). We found that total CD4^+^ T lymphocytes in response to L-mDCs show inhibited proliferation, together with an increase in INF-γ levels, when compared to T-cells stimulated by mDCs. Furthermore, we observed that the proliferation of T-cells stimulated by L-mDCs is recovered to normal levels in the presence of anti-IL-10 antibodies. Taking into account the increased levels of IL10 generated by L-mDCs, we decided to analyze the number of regulatory T-cells (T-regs). We found higher numbers of T-regs, identified as CD4^+^CD25^high^ FoxP3^high^ T-cells, in the co-cultures with L-mDCs than those using mDCs as stimulators.

## Discussion

The data presented in this paper indicate that DCs differentiated from monocytes in the presence of lenalidomide (L-DCs) have a phenotype that could be considered as semi-mature, when compared with control iDCs. The expression of antigen-presenting molecules such as HLA-DR and CD1d was increased in L-DCs. The higher expression of CD1d appears to be controlled at a transcriptional level, since in L-DCs, there is an upregulation of the *CD1D*-encoding mRNA, which is paired to the downregulation of *CD1A* mRNA. The counter-regulation of *CD1A* and *CD1D* expression has been described in several instances and is dependent on the activation of PPARγ (34, 35). The increase of CD1d protein expression on the surface of L-DCs results in a higher ability of NKT cells to lyze autologous L-DCs. It is well known that lenalidomide enhances NKT activation “*in vitro*” and “*in vivo*” (20, 36). Thus, it is possible that a part of the drug’s effect observed in NKT cells could be related to its capacity to increase CD1d expression on antigen-presenting cells. L-DCs exhibit higher amounts of HLA-DR molecules on their surfaces; although there is a slight increase in the mRNA expression of *HLA-DRA* gene, the higher expression of the proteins on the surface of L-DCs is not likely to be transcriptionally controlled since the total levels of HLA-DR proteins, i.e., the intracellular and cell surface levels, were similar in L-DCs and DCs. The upregulation of HLA-DR molecules on the surface of mature DCs is posttranslationally regulated by the cell membrane-bound ubiquitin ligase MARCH-I. This enzyme is downregulated in the DCs during their maturation process (28), resulting in a reduced rate of internalization of HLA-DR molecules present on the surface of mature DCs. In addition, CD83 is upregulated during the maturation process of DCs, and opposes the action of MARCH-I, contributing to an increase in the stability of HLA-DR molecules on the cell membrane (30). The fact that the number of CD83 molecules are dramatically increased on L-DCs, together with the downregulation of MARCH-I may well explain the enhanced expression of HLA-DR that was observed on the L-DC surfaces. In addition, L-DCs exhibit increased levels of additional maturation markers such as CD83 and CD86 on their cell surface. In contrast, when we compared the cases for iDCs and L-iDCs, HLA-class I and CD80 (also markers of DCs maturation) remained at similar levels. Thus, according to their phenotype, L-iDCs could be described as semi-mature DCs; however, this is not the case from a functional point of view. Immature DCs are functionally characterized by their high phagocytic capacity, which decreases after their maturation. L-iDCs behave as normal iDCs with regards to their opsonic phagocytic capacity, indicating that L-iDCs are not functionally semi-mature. Moreover, non-opsonic phagocytosis was more efficient in L-iDCs than in iDCs, and this feature correlates with the expression of DC-SIGN on the cell surface, a molecule typically downregulated on mature DCs. Thus, L-DCs exhibit a unique phenotype and display an enhanced antigen uptake capacity compared to control immature DCs.

With regards to cytokine production, our data indicated a dramatic reduction in the secretion capacity of IL-6 and, especially, TNF-α in L-DCs compared to that in control DCs. This correlates with lower levels of ERK phosphorylation in both L-iDCs and L-mDCs, compared to iDCs and mDCs, respectively, which is in agreement with the role of ERK in the regulation of TNF-α production at both the transcriptional and posttranslational levels (37). These data are consistent with the fact that lenalidomide was originally described as an inhibitor of the production of pro-inflammatory cytokines such as TNF-α, IL-1, and IL-6 (1, 14). On the other hand, L-DCs exhibit an enhanced secretion of IL-10 and IL-12, both of which were abrogated in the presence of a selective inhibitor of Raf-1. Raf-1 can be activated after cross-linking between DC-SIGN and TLR4, which occurs in the presence of certain microbial products such as mycobacterial pathogens, *Candida albicans*, HIV, or measles virus (33). In these cases, Raf-1 acetylates the p65 subunit of the transcription factor NF-kB, after it has been phosphorylated through the TLR-4 signaling pathway, resulting in prolonged and increased transcription of both the *IL-10* and *IL-12* genes (33). In our experiments, we are solely activating TLR4 with LPS. Thus, crosslinking between DC-SIGN and TLR4 should not be expected, at least in control DCs. In contrast, L-DCs exhibit a much higher expression of DC-SIGN than normal iDCs, even after treatment with LPS. In this situation, DC-SIGN might signal the activation of Raf-1. Another possibility is that DC-SIGN signaling could be induced by endogenous ligands such as C1q. It has been recently found that iDCs produce C1q and C1q receptor (C1qR), and that C1q, C1qR, and DC-SIGN can interact, forming a trimolecular complex capable of signaling to activate NF-κB (38), which correlates with the increased NFκB-p65 phosphorylation that we observed in the DCs differentiated in the presence of lenalidomide. The enhanced IL-10 production may explain the fact that allogeneic CD4^+^ T-cells are not fully activated when co-cultured with L-mDCs, since they fail to proliferate in the “*in vitro*” mixed leukocyte reaction assays, although they upregulate the activation marker CD25. Similar findings were observed in the case of memory peptide-specific CD4^+^ and CD8^+^ T-cells. A soluble factor produced by either L-mDCs or the partially activated T-cells appears to be responsible for the inhibition of cell proliferation. Our blocking experiments with specific antibodies strongly suggest that IL-10 is the main factor inhibiting the T-cell proliferation. It is interesting to note that in cultures with L-DCs, there is an increase of regulatory T-cells that may also interfere with the proliferation of their effector T-cell counterparts. The profile of cytokines that we observed in the MLR experiments strongly suggest that L-DCs were able to induce a bias toward a Th1 differentiation of CD4^+^ naïve T-cells, and this is in agreement with their enhanced capability to secrete IL-12. The fact that L-DCs also produce large amounts of IL-10, together with an increase in the numbers of T-regs can explain the safe profile of lenalidomide despite its powerful capability to activate conventional T-cells as well as NKT cells that could otherwise produce excessive amounts of proinflammatory cytokines. In this regard, it has been recently shown that lenalidomide can enhance the “*in vitro*” recovery of monocyte-derived DCs generated from MM patients (2, 5, 24, 39).

The data presented in this manuscript indicate that DCs differentiated from monocytes obtained from healthy blood donors in the presence of lenalidomide display a semi-mature phenotype. However, from the functional point of view, these cells are more phagocytic, and when they are stimulated by LPS, they produce lesser amount of proinflammatory cytokines, and display an enhanced capacity to polarize Th1 responses, while they enhance IL-10 secretion and Treg expansion, which could contribute to regulate excessive, potentially harmful, activation of effector T-cells. These features might be useful to promote the development or the potentiation of safe Th1 immune responses in different pathologies such as cancer or infectious diseases.

## Author Contributions

EM-N designed the study, interpreted the data, and wrote the manuscript. JL-R designed and performed experiments, interpreted the data, and contributed to the writing of the manuscript. JL-B, BeA, I R-A, and BáA helped to perform experiments. SS-R and BM-A contributed to data analysis and interpretation. MM interpreted data and contributed to the writing of the manuscript.

## Conflict of Interest Statement

The authors declare that the research was conducted in the absence of any commercial or financial relationships that could be construed as a potential conflict of interest.
