# Supplementary material for: Monocyte-Derived Dendritic Cells Differentiated in the Presence of Lenalidomide Display a Semi-Mature Phenotype, Enhanced Phagocytic Capacity, and Th1 Polarization Capability
Source: Front Immunol. 2018 Jun 13;9:1328. doi: 10.3389/fimmu.2018.01328 (PMC6008535; doi:10.3389/fimmu.2018.01328)
Supplement: Supplementary file 1 [file Image_1.PDF]

Supplementary Figure 1

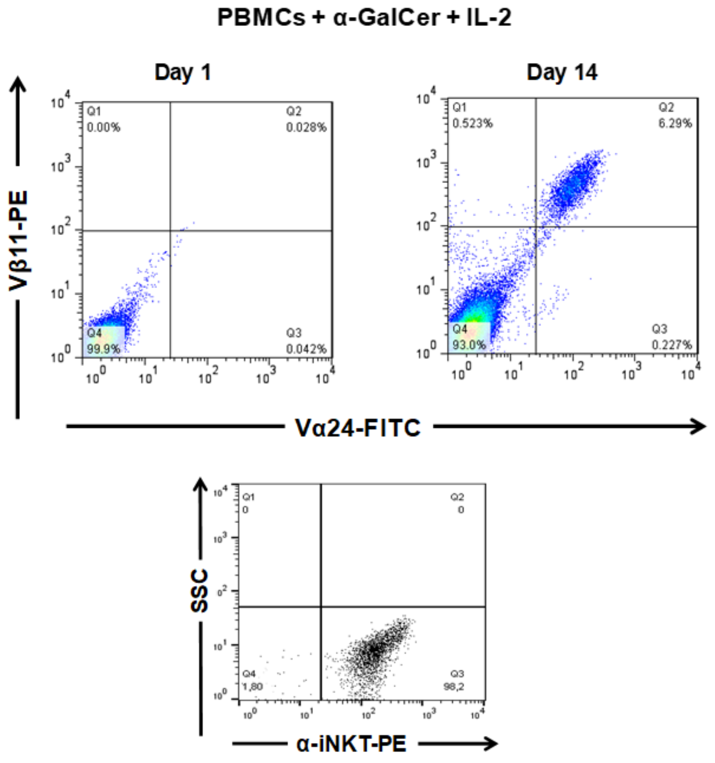

Supplementary Figure 1. iNKT expansion and isolation.

PBMCs were cultured in RPMIc with IL-2 and α-GalCer for two weeks. iNKTs were then isolated using Miltenyi's microbead isolation kit. Purity of the cells was assessed by staining with the anti-iNKT antibody.
